# Profiling the gut structure and microbiota, and identifying two dominant bacteria belonging to the *Weissella* genus in mandarin fish (*Siniperca chuatsi*) fed an artificial diet

**DOI:** 10.3389/fmicb.2024.1486501

**Published:** 2024-11-29

**Authors:** Jiayu Wang, Yaotong Hao, Lihan Zhang, Xiaowei Gao, Yihuan Xu, Jiangjiang Wang, Fazhan Hanafiah, Waiho Khor, Yanfeng Sun, Chengbin Wu

**Affiliations:** ^1^Ocean College, Hebei Agricultural University, Qinhuangdao, Hebei, China; ^2^Hebei Key Laboratory of Nutritional Regulation and Disease Control for Aquaculture, Qinhuangdao, Hebei, China; ^3^Higher Institution Center of Excellence (HICoE), Institute of Tropical Aquaculture and Fisheries, University Malaysia Terengganu, Kuala Terengganu, Terengganu, Malaysia

**Keywords:** *Siniperca chuatsi*, artificial diet, gut histology, gut microbiota, *Weissella*

## Abstract

Mandarin fish (*Siniperca chuatsi*) fed with an artificial diet is progressively gaining popularity, which is important for reducing product prices and resource consumption. However, food is the decisive factor of intestinal microbes, and the profound effects of change in their feeding habit on intestinal microbes of mandarin fish have not been revealed. In the present study, live bait fish and artificial diet were used to feed mandarin fish for 8 weeks to study the effect of different feeding habits on the histology, microbiota structure and dominant bacteria of gut in mandarin fish. The results indicated that feeding with the artificial diet significantly increased the intestinal villi height and muscular thickness in the hindgut of mandarin fish. In addition, the microbiota results showed that there were significant differences of beta diversity of gut microbiota in mandarin fish fed with different diets. At the genus level, feeding artificial diets significantly increased the abundance of *Weissella* in the mandarin fish gut. Furthermore, two *Weissella* bacteria were identified and characterized from the midgut contents of mandarin fish fed with artificial diet. Based on 16S rRNA gene sequencing, nine strains were assigned as *Weissella confusa* (RM125), and one as *Weissella cibaria* (SJ548). Biochemical analyses based on the VITEK2 method revealed a pattern of metabolic activities against *W. confusa* RM125 and *W. cibaria* SJ548, with 13 positive and 29 negative results, respectively. *W. confusa* RM125 and *W. cibaria* SJ548 exhibited sensitivities toward a variety of pathogens, including *V. harveyi*, *S. aureus* and *V. parahaemolyticus*, *E. coli*, *A. hydrophila*, *S. enterica*, *V. anguillarum*, and *V. alginolyticus*, indicating potential probiotics. Therefore, our results confirmed that the transformation of feeding habit altered the structure, microbial composition and dominant bacteria in gut of mandarin fish, and provided evidence that *Weissella* might play a crucial role in the digestion and absorption of artificial diet in mandarin fish.

## 1 Introduction

Mandarin fish (*Siniperca chuatsi*) is a special carnivorous fish species in China, with an annual culture production exceeding 370,000 tons since 2020, further reaching 470,000 tons in 2023. Traditionally, mandarin fish relies on live bait fish since their first-feeding stage owing to their unique preference of feeding habits (Shen et al., 2023). Increasing studies have shown that mandarin fish have a certain degree of adaptability to artificial diet feeding (Li et al., 2019). Besides, the higher survival rates and lower bait coefficients have suggested that the artificial feeds can be successfully used for intensive commercial culture of mandarin fish (Shen et al., 2021; Li et al., 2023; Dou et al., 2018; Zhang et al., 2024). Notably, domestication and cultivation of mandarin fish by artificial diet feeding can achieve great profitability by reducing the demand for live bait fish. However, there are no large-scale domesticated commercial mandarin fish being sold in the market, which highlights that they remain immature. In recent years, researchers have conducted a series of studies in the elucidation of the regulation mechanism of feeding habit of mandarin fish, which was related to appetite and digestive tract structure as well as the feed palatability. Still, the effects of artificial diet on the digestive system of domesticated mandarin fish remains incompletely elucidated.

The gut is an important part of the digestive system, and the microorganisms colonizing the gut are involved in the host’s food digestion, nutrient absorption, energy metabolism, growth and development, and immune regulation, which are essential for maintaining fish health (Vine et al., 2004; Brugman et al., 2018; Shen et al., 2020; Vargas-Albores et al., 2021). Previous studies have shown that diets and gut histology are highly correlated, and the relatively complex gut structure of mandarin fish promotes nutrient absorption (Li et al., 2024). Increasing studies have discovered that dietary modifications can lead to shifts in the gut microecology of aquatic animals (Ma et al., 2021; Wei et al., 2023; Shi et al., 2024; Huang et al., 2024). Meanwhile, the composition of intestinal microbes is intimately associated with the activities of digestive enzymes, which are capable of enhancing the host’s metabolism by converting indigestible compounds into utilizable metabolites for the host (Kotzamanis et al., 2007). Therefore, more and more beneficial microbes were isolated and applied in the aquaculture not only for their nutritive effect in food utilization but also for their probiotics effects (Ray et al., 2012; Huy et al., 2020). It is worth noting, these probiotics exhibit robust antimicrobial properties, as colonized microbiota suppresses pathogens through nutrient competition and safeguard the host from infection by directly competing with pathogens via their ecological niche occupation (Liu et al., 2021). For instance, the *W. confusa* N17 isolated from the gut of loach (*Misgurnus anguillicaudatus*) demonstrated significant destructive effects on *Aeromonas hydrophila* TH0406 and TPS (Yang et al., 2023). Collectively, to enhance various aspects such as fish feeding efficiency, growth, immunity, and overall survival rates, the incorporation of probiotics into artificial feeds or water were widely used in aquaculture (Tao et al., 2024; Jin et al., 2024; Vargas-González et al., 2024). However, there is a scarcity of research examining the alterations in gut histology and intestinal microbiota of mandarin fish following a dietary transition.

To improve the efficiency of domestication of mandarin fish adapting to artificial diets, several investigations have focused on the uncovering mechanisms from inherited differences. However, few studies focused on the gut microbes related to domestication of mandarin fish. Therefore, in our study, we investigated the effect of artificial diets on the gut histology as well as the gut microbiota of mandarin fish. Most importantly, we aimed to isolate and obtain potential probiotics adapted to the gut environment of mandarin fish that were fed artificial diets. This is of great significance to prove effects of exogenous factors on the feed preference of mandarin fish and provides a novel insight for further studies on artificial diet domestication of mandarin fish.

## 2 Materials and methods

### 2.1 Ethics statement

All animal experimental procedures were performed in accordance with the Regulations for the Administration of Affairs Concerning Experimental Animals approved and authorized by the State Council of the People’s Republic of China and the Animal Ethics Committee of Hebei Agricultural University (No. 2023036). Fish were sacrificed, and all efforts were exerted to minimize suffering.

### 2.2 Experimental design

**Figure 1:**
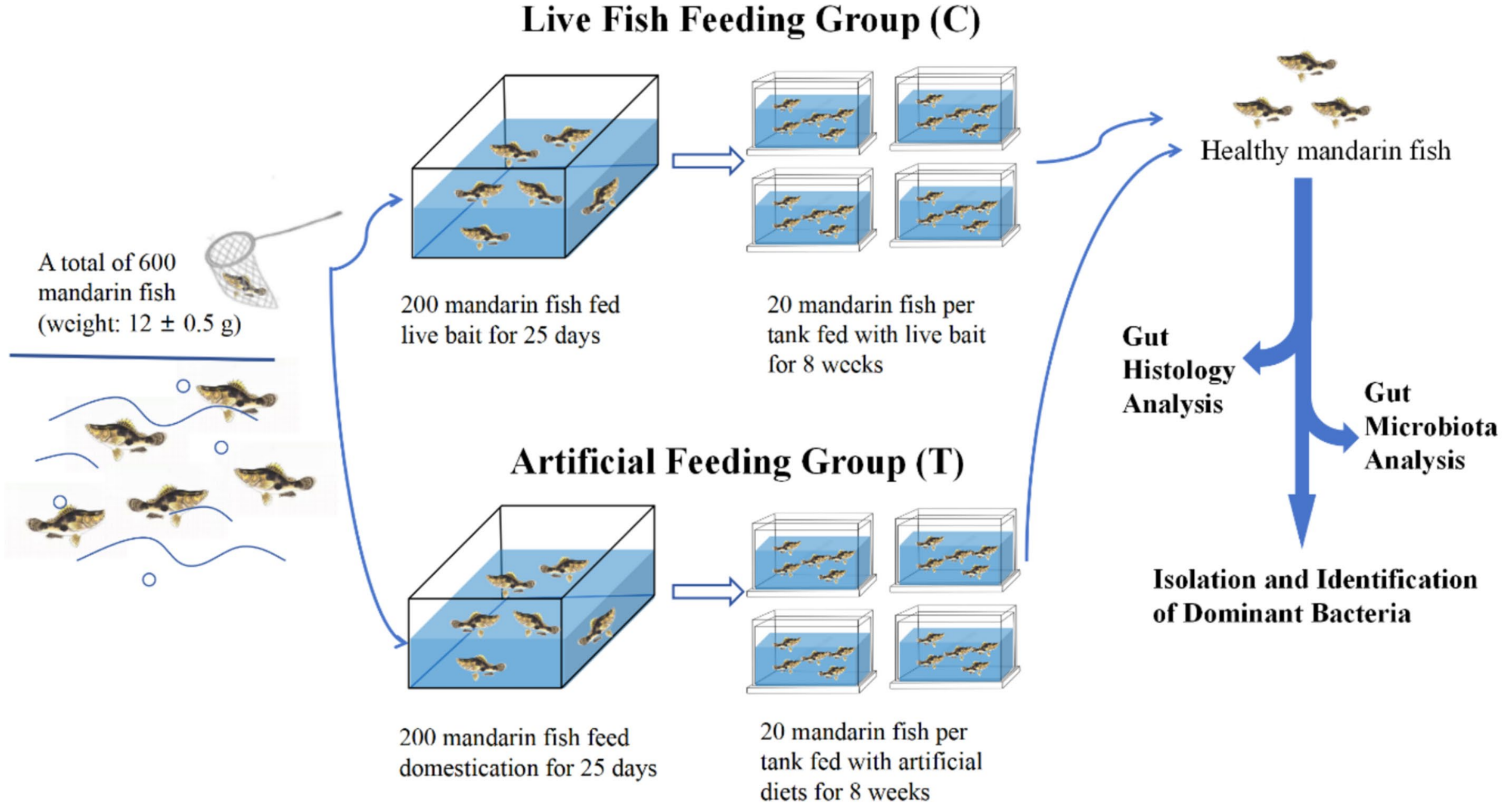
The experimental step of the present study.

The experimental step was shown in Figure 1. A total of 600 healthy mandarin fish (weight: 12 ± 0.5 g) were stocked into indoor canvas tanks for 7 days. During the acclimation period, the fish were fed twice a day (at 06:00 and 18.00 h) with live India mrigal (*Cirrhinus mrigala*) as bait. Afterwards, a total of 200 uniform-sized healthy fish fed with live bait fish, designated as the live fish feeding group (Control, C), and another 200 healthy mandarin fish were taken for feeding artificial diets for 25-day domestication period (Liang et al., 2001; Wang et al., 2024), designated as the artificial feeding group (Treatment, T). Subsequently, 80 uniform-sized fish from C and T groups, respectively, were randomly distributed into four tanks with 20 fish each tank, and fed with live bait and artificial diet for 8 weeks, respectively. The nutrient composition of artificial diets for mandarin fish is shown in Supplementary Table S1. The water quality of each tank was maintained at an optimal range of physical parameters: temperature (28.0 ± 2.0°C), pH (7.5 ± 0.5), ammonia-nitrogen (0.2 ± 0.05 mg/L), and dissolved oxygen (5.0 ± 0.5 mg/L) during the experimental period.

After 8-week feeding, three individuals from each tank were first placed in an ice-water mixture for 20 min. When they stopped swimming and their gills ceased to move, the fish were transferred to a sterile white porcelain dish, rinsed with sterile water, and then sterilized with 75% alcohol. Subsequently, the guts from each fish were dissected out, and the foregut, midgut, and hindgut tissues were also collected and fixed in Bouin’s fixative for histological observation. The contents of the midgut from three parallel fish in each tank were collected and stored at −80°C for microbiota analysis, as well as the isolation and identification of dominant bacteria.

### 2.3 Histological observation of the gut

Histological observation of the gut from *S. chuatsi* was conducted based on the method described in Xu et al. (2022). Briefly, the gut tissue underwent a series of procedures including dehydration with alcohol, immersion in paraffin, and staining with hematoxylin and eosin (H&E). The gut tissue morphology was then observed and photographed under a microscope (BX40F4, Olympus, Tokyo, Japan). ImageJ software was utilized to measure the intestinal villus height and the intestinal muscular thickness.

### 2.4 Gut microbiota analysis

#### 2.4.1 DNA extraction and PCR sequencing

The bacterial genomic DNA was extracted from the gut samples and artificial diet using a DNA rapid extraction kit (TransGen, Beijing). Subsequently, the extracted DNA was amplified by PCR with universal primers (338F: 5′-ACTCCTACGGGAGGCAGCAG-3′ and 806R: 5′-GGACTACHVGGGTWTCTAAT-3′). 1% agarose gel electrophoresis was conducted to ensure the quality of the extracted DNA. All samples were sequenced using the Illumina NovaSeq 6,000 platform.

#### 2.4.2 Sequencing data analysis

The sequencing data analysis of 16S rRNA was performed based on the method described in Hao et al. (2023). Briefly, the quality of sequencing data was assessed using Trimmomatic and Cutadapt, followed by the calculation of diversity indices and evaluation of sampling depth. The Ace indices, Chao1 indices, Shannon indices, Simpson indices, and operational taxonomic units (OTUs) were used to assess the Alpha diversity of mandarin fish’s gut microbiota. The coverage of every sample library was employed to evaluate the validity of the sequencing results. Principal coordinates analysis (PCoA) was used to assess the beta diversity of the gut microbiota. Each microbiota’s relative abundance was computed at the phylum and genus levels. The evolutionary relationship between the samples was examined by UniFrac. Welch’s *t*-test and the non-parametric factorial Kruskal-Wallis (KW) sum-rank test were used to compare the two groups. The figure of linear discriminant analysis effect size (LEfSe) was made based on linear discriminant analysis (LDA) to calculate the contribution of each component’s richness to the variation. Hierarchical clustering analysis was performed based on the binary-jaccard distance matrix. The tree structure diagram was constructed according to the Unweighted Pair-Group Method with Arithmetic Mean (UPGMA) algorithm. Then, the functional category abundance information of the microbial community was determined by analyzing the KEGG database using the PICRUSt2 microbial community metabolic function prediction tool.

### 2.5 Isolation and identification of *Weissella* bacteria

#### 2.5.1 Isolation of *Weissella* bacteria

Based on the 16S rRNA sequencing data, *Weissella* were the dominant bacteria in the gut of mandarin fish fed an artificial diet. To this aim, *Weissella* bacteria were isolated and identified from gut in mandarin fish.

The gut contents from Group T were rinsed with 1 mL of sterilized saline. The resultant stock solution was collected and diluted to gradients of 10^−1^, 10^−3^, and 10^−5^. Subsequently, 0.1 mL of liquid from each dilution gradient was inoculated onto De Man, Rogosa, and Sharpe (MRS) solid medium supplemented with 0.2 g/L vancomycin (Kumar et al., 2011). The samples were then incubated at a constant temperature of 30°C for 12 h until colonies appeared. Afterwards, single colonies were randomly selected for transplantation and streaked onto fresh medium three times to ensure the purification of the samples.

#### 2.5.2 Molecular identification of *Weissella* strains

The genomic DNA of dominant bacteria was extracted using chloroform method (Sambrook and Russell, 2001), then amplified with primers (27F: 5′-AGAGTTTGATCMTGGCTCAG-3′, and 1492R: 5′-GGTTACCTTGTTACGACTT-3′). After verification via a 1.0% agarose gel, the amplified products were sent to Beijing Zhixu Biotechnology Co., Ltd. for sequencing. The isolated bacteria’s 16S rRNA sequences were analyzed using the National Center for Biotechnology Information (NCBI) database for BLAST analysis. MEGA 11 software and the neighbor-joining method were employed to construct the phylogenetic tree, as reported by Zhang et al. (2016).

#### 2.5.3 Morphological observation, biochemical characterization of *Weissella* bacteria

The shape and hue of the separated colonies were examined using a scanning electron microscope. The individual colonies were selected for Gram staining examinations, based on the Stain Kit (BKMAMLAB, Changde, China). The pure colonies were cultivated in MRS broth for 12 h at 30°C and 200 rpm. After reaching the exponential phase (OD_600nm_ = 0.5 ~ 0.7), the strains were rinsed in PBS buffer (pH 7.4), fixed in 2.5% glutaraldehyde, dried using an alcohol gradient, and resuspended in isoamyl acetate. Then, they were ready for morphological analysis using a scanning electron microscope. The biochemical characterization of bacterial cultures was evaluated using VITEK2 system (Ligozzi et al., 2002). The bacterial species was identified based on the observed patterns of biochemical reactions.

#### 2.5.4 Antibacterial activity of *Weissella* strains

Antibacterial activity was determined using the diffusion method, as described in Yadav and Tiwari (2023). The eight indicator bacteria obtained from Hebei Key Laboratory of Nutritional Regulation and Disease Control for Aquaculture, including *Escherichia coli*, *Aeromonas hydrophila*, *Staphylococcus aureus*, *Salmonella enterica*, *Vibrio anguillarum*, *V. harveyi*, *V. parahaemolyticus*, and *V. alginolyticus*, were selected for measure the antibacterial activity of dominant strains. The selected *Weissella* strains were cultured in MRS broth until their OD_600 nm_ reached 0.7. In brief, the indicator bacteria (1 × 10^8^ CFU/mL) were spread onto LB solid medium with Oxford cups containing three wells filled with *Weissella* strains (OD600 nm = 0.7) and one well filled with distilled water as a control. The results were interpreted as follows: DIZ < 15.00 mm indicated insensitivity (no significance, ns); 15.00 mm < DIZ ≤ 16.00 mm indicated low sensitivity (+); 16.00 mm < DIZ ≤ 17.00 mm indicated moderate sensitivity (++); and DIZ > 17.00 mm indicated high sensitivity (+++).

### 2.6 Statistical analysis

SPSS 26.0 was used to perform statistical analysis on the experimental data, and GraphPad Prism 7.0 was employed for data visualization. The results were expressed as the mean with standard error of the mean (mean ± SEM). A Student’s *t*-test (for independent samples) was utilized for comparisons between two groups. Significant differences were set at *p* < 0.05 (*), *p* < 0.01 (**), *p* < 0.001 (***), or indicated no significant difference (ns).

## 3 Results

### 3.1 Histological observation of the gut

**Figure 2:**
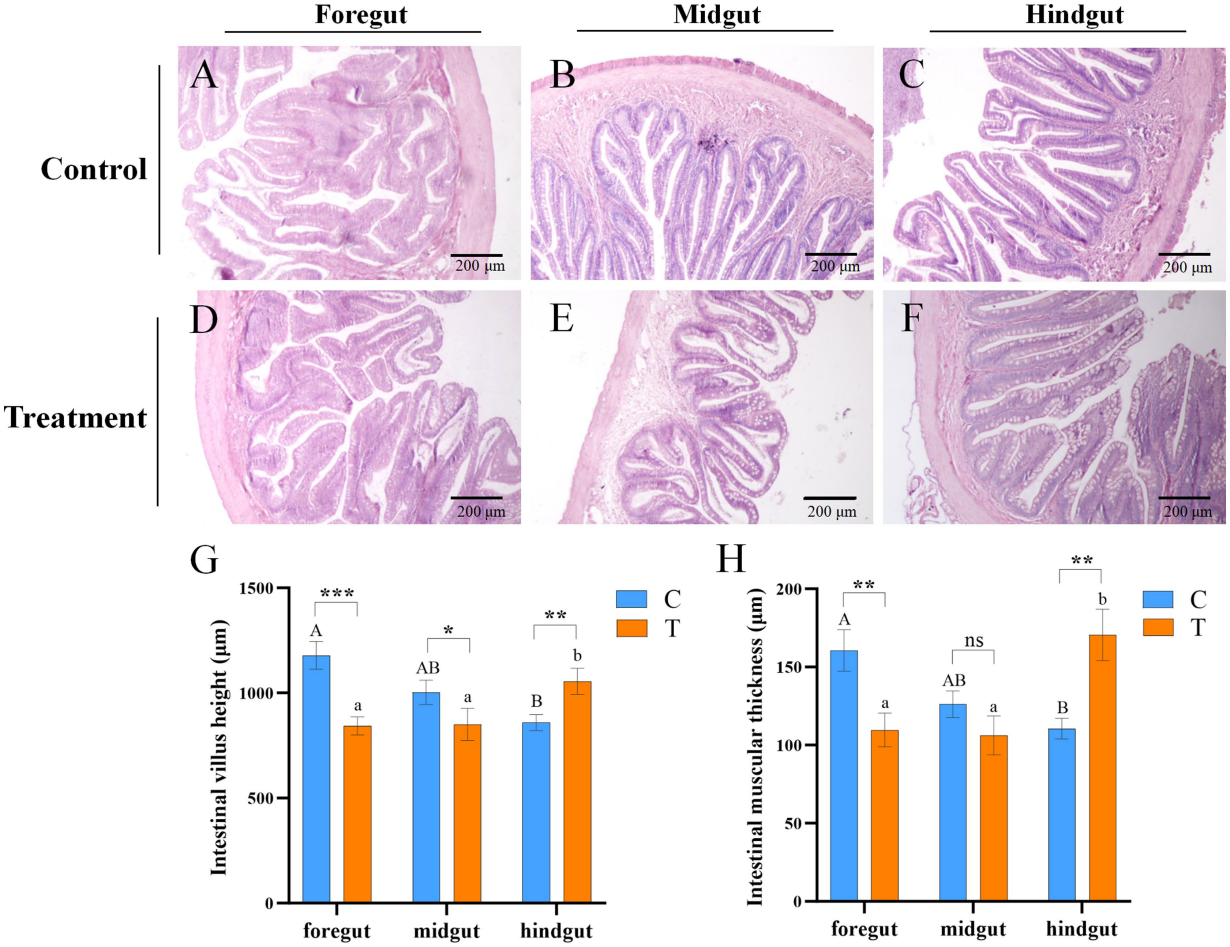
Microscopic images of intestinal tissue sections of mandarin fish fed for 8 weeks in different feeding groups (C and T). **(A–F)** HE staining; Scale bar: 200 μm; Magnification: 100×. **(A)** Foregut, **(B)** midgut, and **(C)** hindgut of mandarin fish fed with live bait (Group C). **(D)** Foregut, **(E)** midgut, and **(F)** hindgut of mandarin fish fed with artificial diet (Group T). **(G)** The intestinal villi height of mandarin fish in different feeding groups (C and T). **(H)** The intestinal muscular thickness of mandarin fish in different feeding groups (C and T). Data were presented as mean ± SEM (*n* = 3). *p < 0.05, a significant difference; **p < 0.01, extremely significant difference; ns: no significant difference. H.E., hematoxylin and eosin staining; C, live fish feeding group; T, artificial feeding group.

After 8-week feeding, the changes in the guts of the C and T groups were carefully examined. As shown in Figures 2A–F, the structure of the gut from C and T groups exhibited regular and compact arrangement, with orderly aligned intestinal villi and intact structure.

The heights of intestinal villi and thickness of the intestinal muscular in Group C were gradually decreased from foregut to hindgut, but those were incrementally increased in Group T (Figures 2G,H). Compared to Group C, the heights of intestinal villi were significantly reduced in the foregut and midgut (*p* < 0.05), but significantly elevated in the hindgut of Group T (*p* < 0.05) (Figure 2G). The thickness of intestinal muscular was significantly decreased in foregut, but increased in hindgut of Group T, compared with Group C (*p* < 0.05) (Figure 2H).

### 3.2 Gut microbiota analysis

#### 3.2.1 16S rRNA sequencing results

In the present study, we obtained a total of 678,375 quality-controlled reads and 642,011 effective reads based on NovaSeq Illumina-based paired-end amplicon 16S rRNA gene sequencing (V3–V4) (Supplementary Table S2). A total of 2,601 operational taxonomic units (OTUs) were identified by removing background noise and performing species annotation, representing the diversity of bacterial species present in the samples. The dilution curves of samples tended to be flat and were close to the plateau, indicating that the current sequencing depth was sufficient to capture the bacterial community’s biological information (Supplementary Figure S1).

#### 3.2.2 Diversity index analysis

**Figure 3:**
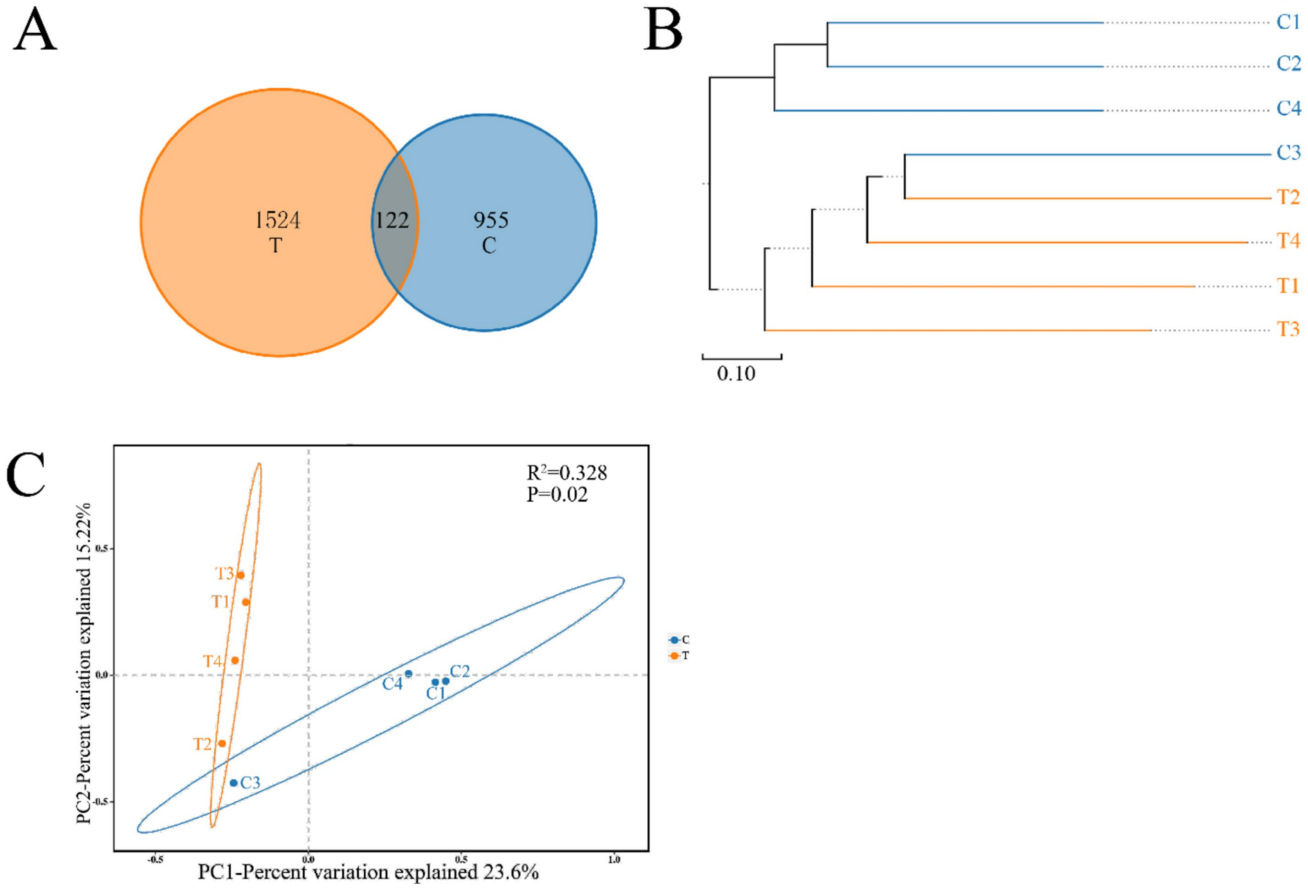
Venn diagram analysis **(A)** depicting the numbers of shared and unique OTUs of mandarin fish gut microbial populations in different feeding groups (C and T). The beta diversity distance matrix **(B)** of gut bacteria samples was analyzed using hierarchical clustering, and a tree structure diagram was subsequently constructed using the UPGMA algorithm. PCoA **(C)** based on the weighted UniFrac distance of the gut bacterial communities of mandarin fish in different feeding groups (C and T). C, live fish feeding group; T, artificial feeding group.

The Chao 1, ACE, Shannon, and Simpson indices were employed to assess the diversity and richness of the gut microbiota in two distinct groups of mandarin fish (Supplementary Table S2). Notably, although the average values for the Ace, Chao1, Shannon, and Simpson indices were higher in Group T compared with Group C, there were no significant differences across all four metrics (*p* > 0.05) (Supplementary Figure S2). A Venn diagram was constructed to identify the core and different Operational Taxonomic Units (OTUs), revealing that a total of 122 OTUs shared between Group T and C (Figure 3A). The hierarchical cluster analysis was conducted on the beta diversity distance matrix of the gut microbiota, resulting in a tree structure diagram generated by the UPGMA algorithm (Figure 3B). Principal Coordinates Analysis (PCoA) was also employed to explore differences in microbial community composition (Figure 3C). In detail, PC1 accounted for 23.6% of the total bacterial community variation, while PC2 explained 15.22% (Figure 3C). Significant differences were observed between the gut bacterial communities of mandarin fish fed live bait and those fed an artificial diet (*p* < 0.05) (Figure 3C).

#### 3.2.3 Community composition and difference analysis

**Figure 4:**
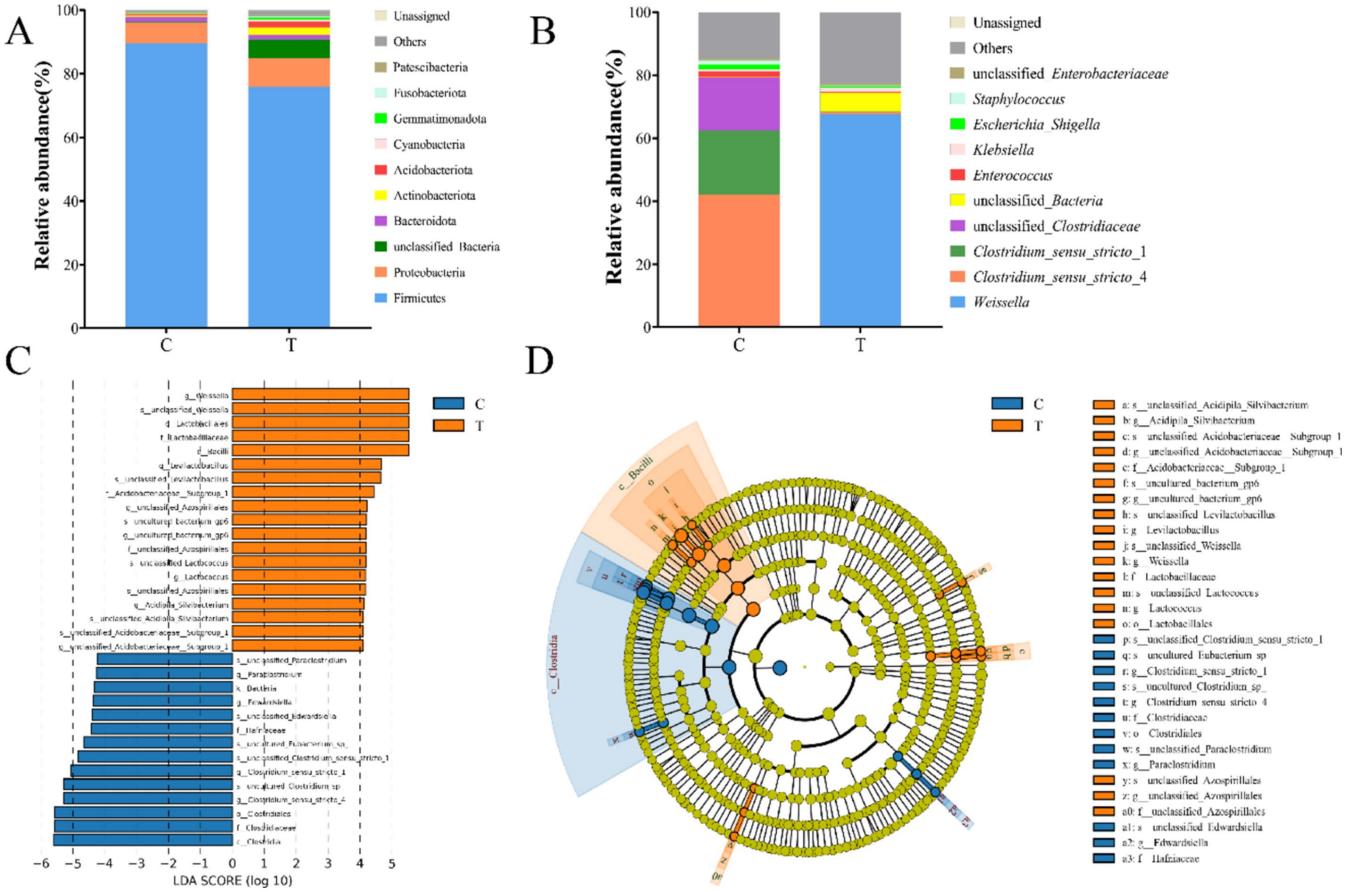
The community composition of gut microbiota of mandarin fish in different feeding groups (C and T) at the level of phylum **(A)** and genus **(B)**. Difference scores of LEfSe **(C)** of the gut microbial of mandarin fish. LDA scores threshold >4. Evolutionary branching diagram of LEfSe analysis **(D)** of the gut microbial of mandarin fish. C, live fish feeding group; T, artificial feeding group.

Distinct community structures of gut microbiota were observed in mandarin fish fed with live bait and artificial diet. To eliminate the effect of artificial diet on gut microbiota, we detected the abundance of species at the level of bacterial genera in the artificial diet (Supplementary Table S3). As shown in Figure 4A, Firmicutes was the most abundant bacteria at the phylum level in Group C, followed by Proteobacteria and Bacteroidota. In addition, Firmicutes was dominant, followed by Proteobacteria, Actinobacteota, Acidobacteriota, and Bacteroidota in Group T (Figure 4A). At the genus level, *Clostridium_sensu_stricto*_4 (41.98%) and *Clostridium_sensu_stricto_*1 (20.45%) were the dominant bacteria in Group C. However, *Weissella* (67.70%) was the most common bacteria in Group T (Figure 4B). Notably, there was a significant difference in the relative abundances of *Clostridium*_*sensu*_*stricto*_4 (*p* < 0.05), *Clostridium*_*sensu*_*stricto*_1 (*p* < 0.05), and *Weissella* (*p* < 0.05) between the two groups. LEfSe analyses showed a higher abundance of 14 and 19 taxa in Group C and T, respectively (LDA score > 4.0) (Figure 4C). As shown in Figure 4D, the biomarkers of Group C consisted of *Clostridium_sensu_stricto_*4, *Clostridium_sensu_stricto*_1, *Paraclostridium*, E*dwardsiella*, Clostridiaceae, Hafniaceae, Clostridiacles, and Clostrdia. Additionally, the biomarkers of Group T were composed of *Lactococcus*, *Weissella*, *Evilactobacillus*, A*cidipilasilvibacterium*, Acidobacteriaceae, Lactobacillales, and Bacilli.

#### 3.2.4 Functional prediction

**Figure 5:**
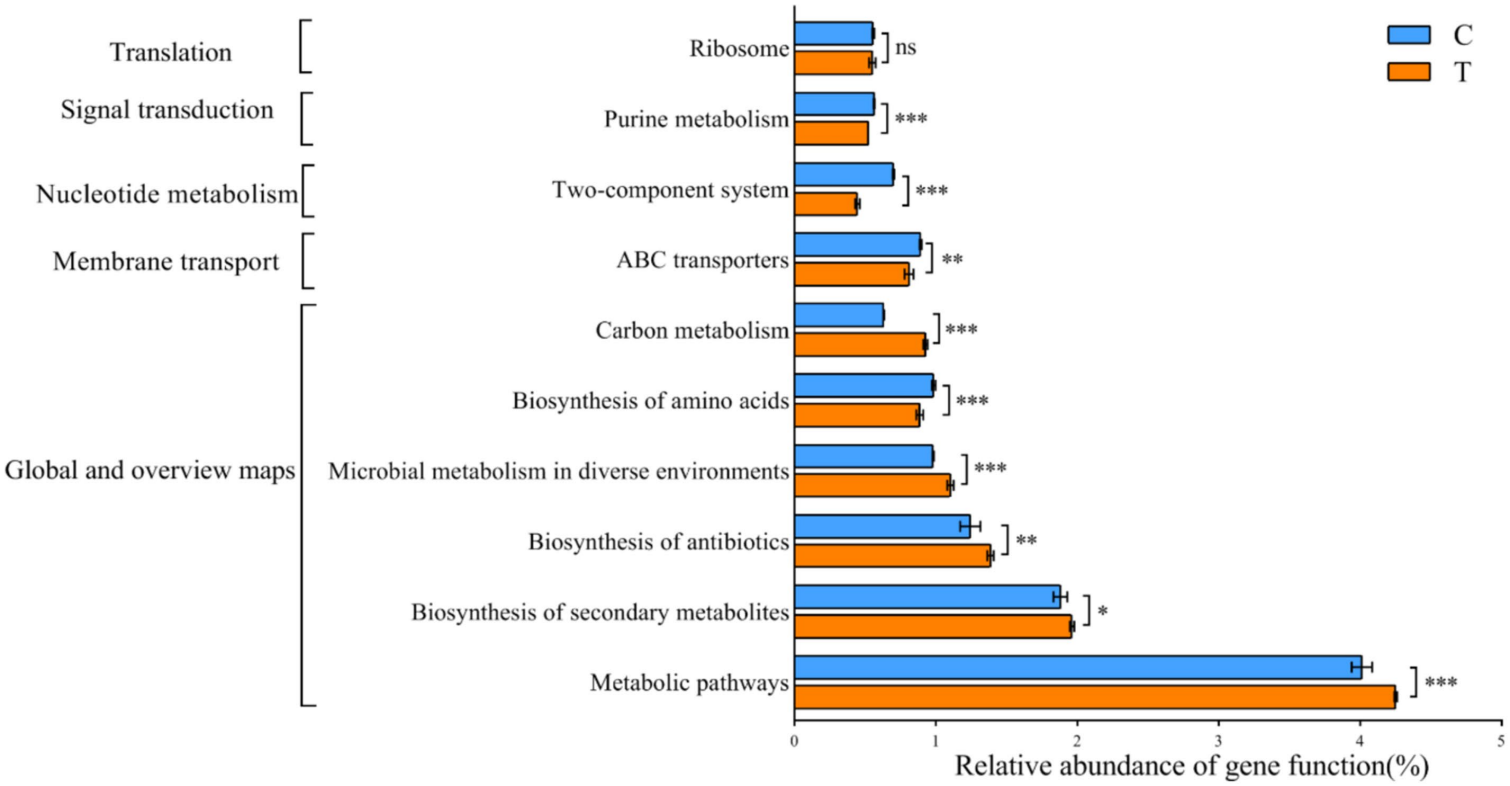
Predicted function of KEGG metabolic pathway in the microbial communities of gut in mandarin fish from different feeding groups (C and T). Data were presented as mean ± SEM. *p < 0.05, significant difference; **p < 0.01, extremely significant difference; ns, no significant difference. C, live fish feeding group; T, artificial feeding group.

The KEGG database was utilized for functional prediction of 16S rRNA gene derived from the gut microbiota of mandarin fish. In the present study, 341 metabolic functional pathways were identified, and the genes of the gut microbiota were categorized into six primary metabolic pathways, involved in metabolism, genetic information processing and environmental information processing (Supplementary Table S4). PICRUSt2 analyses revealed that, despite being from different feeding groups (C and T) of the mandarin fish exhibited similar gene functions (Figure 5). There were significant differences in the abundance of nine pathways including metabolic pathways, biosynthesis of secondary metabolites, and microbial metabolism in diverse environments (*p* < 0.05) (Figure 5). The relative abundance of several pathways in Group T were higher than that in Group C, including biosynthesis of secondary metabolites, biosynthesis of antibiotics, microbial metabolism in diverse environments, and carbon metabolism (*p* < 0.05) (Figure 5).

### 3.3 Isolation of potential bacteria

#### 3.3.1 Preliminary observations and identification of the *Weissella* bacteria

**Figure 6:**
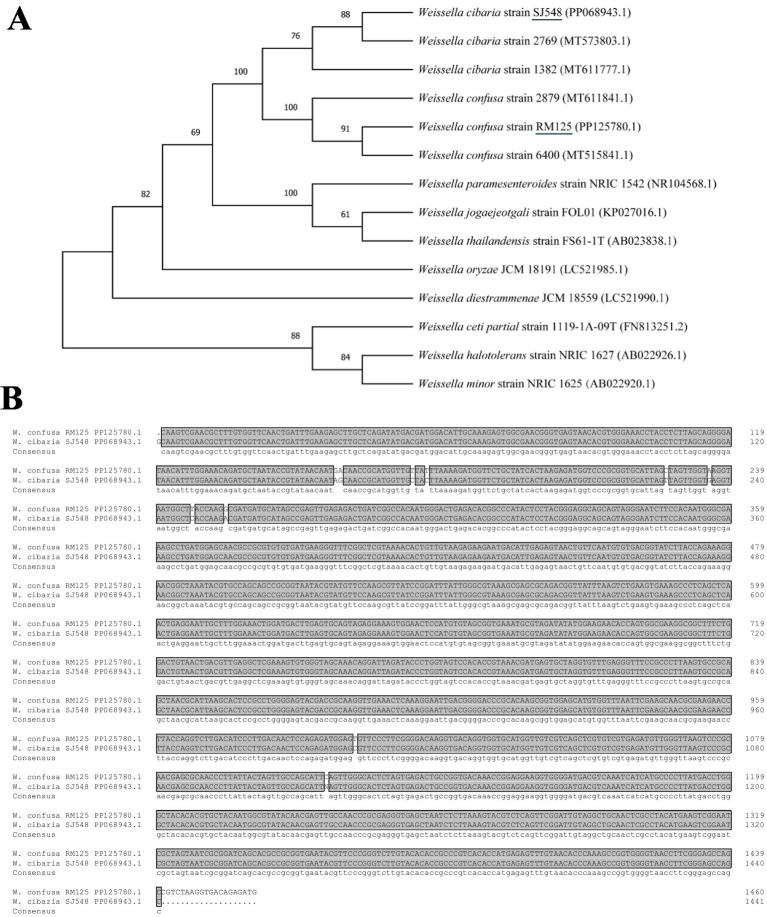
Phylogenetic trees **(A)** based on the 16S rRNA sequences of *Weissella confusa* RM125 and *Weissella cibaria* SJ548 and other *Weissella*, constructed by neighbor-joining method. Numbers at the branches represented the bootstrap support values. ClustalX alignment **(B)** of the 16S rRNA sequences of *Weissella confusa* RM125 and *Weissella cibaria* SJ548. The identical sequences were shaded dark gray. Accession numbers: *Weissella cibaria* strain SJ548, PP068943.1; *Weissella cibaria* strain 2,769, MT573803.1; *Weissella cibaria* strain 1,382, MT611777.1; *Weissella confusa* strain 2,879, MT611841.1; *Weissella confusa* strain RM125, PP125780.1; *Weissella confusa* strain 6,400, MT515841.1; *Weissella paramesenteroides* strain NRIC 1542, NR104568.1; *Weissella jogaejeotgali* strain FOL01, KP027016.1; *Weissella thailandensis* strain FS61-1 T, AB023838.1; *Weissella oryzae* JCM 18191, LC521985.1; *Weissella diestrammenae* JCM 18559 (LC521990.1); *Weissella ceti* partial strain 1,119-1A-09 T, FN813251.2; *Weissella halotolerans* strain NRIC 1627, AB022926.1; *Weissella minor* strain NRIC 1625, AB022920.1.

Ten strains of bacteria from punctate colonies were isolated for 16S rRNA sequencing. These sequences were blasted in the NCBI database, revealing that nine strains shared identical sequences, while one strain was different. The phylogenetic tree indicated that the nine strains were *Weissella confusa* and one strain was *Weissella cibaria*, designated as *W. confusa* RM125 (NCBI accession number: PP125780.1) and *W. cibaria* SJ548 (NCBI accession number: PP068943.1), respectively (Figure 6A). *W. confusa* RM125, *W. confusa* 2,879 and *W. confusa* 6,400 clustered together based on their similarity of 97.75% (Supplementary Figure S3). Moreover, *W. cibaria* SJ548, *W. cibaria* 1,382 and *W. cibaria* 2,769 gathered into a cluster with their similarity of 99.17% (Supplementary Figure S4). As shown in Figure 6B *W. confusa* RM125 and *W. cibaria* SJ548 shared a similarity of 97.81%.

#### 3.3.2 Morphological observation and biochemical identification

**Figure 7:**
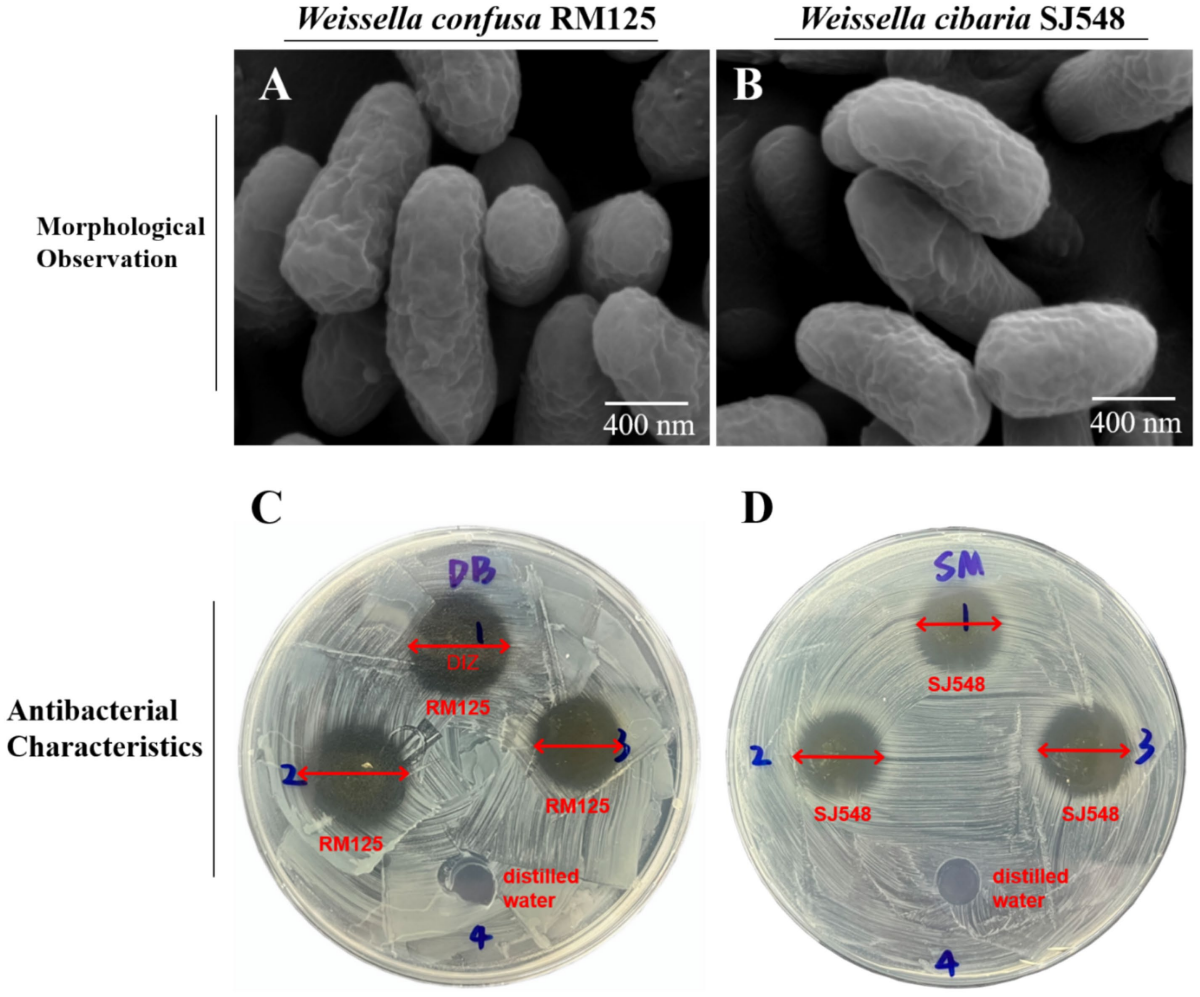
Morphological observation of *Weissella confusa* RM125 **(A)**, *Weissella cibaria* SJ548 **(B)** using the scanning electron microscope. Scale bar: 400 nm. The schematic diagram of antibacterial characteristics of *W. confusa* RM125 **(C)** and *W. cibaria* SJ548 **(D)** using Oxford cups. Well 1–3 were filled with *W. confusa* RM125 **(C)** or *W. cibaria* SJ548 **(D)**, and Well 4 with distilled water. The plates were examined for the diameter of inhibition zone (DIZ). The results were shown as DIZ < 15.00 mm, insensitivity, no significance (ns); 15.00 mm < DIZ ≤ 16.00 mm, low sensitivity, +; 16.00 mm < DIZ ≤ 17.00 mm, moderate sensitivity, ++; DIZ > 17.00 mm, high sensitivity, +++.

As exhibited in Figure 7, the images from the scanning electron microscope revealed that the cells of *W. confusa* RM125 and *W. cibaria* SJ548 were well organized, displaying rod-like or double rod-like structures with a flat surface and absent of flagella. Specifically, *W. confusa* RM125 had dimensions of approximately 1.15 μm in length and 0.46 μm in width (Figure 7A), while *W. cibaria* SJ548 measured approximately 1.59 μm in length and 0.47 μm in width (Figure 7B).

Biochemical analyses using the VITEK2 method revealed a pattern of metabolic activities against *W. confusa* RM125 and *W. cibaria* SJ548, with 13 positive and 29 negative results, respectively (Supplementary Table S5). Both *W. confusa* RM125 and *W. cibaria* SJ548 strains could utilize N-acetyl-D-glucosamine (NAG), D-mannose (dMNE), Saccharose (SAC) and D-maltose (dMAL) as carbon sources and produce the enzymes including Arginine dihydrolase 1 (ADH1) and Arginine dihydrolase 2 (ADH2). Both these strains were tolerant to Novobiocin (NOVO), Optokhin (OPTO), O129R (O/129) and Bacitracin (BACI), and could grow in the presence of 6.5% NaCl. In contrast, neither strain was able to metabolize Amygdalin (AMY), d-ribose (dRIB), D-raffinose (dRAF), Cyclodextrin (CDEX), D-sorbitol (dSOR), Lactose (LAC), D-mannitol (dMAN), Salicin (SAL), Methyl-B-D-glucopyranoside (MBdG), D-trehalose (dTRE), *α*-glucosidase (AGLU), D-galactose (dGAL), and Pullulanase (PUL). Besides, neither strain could produce Leucine arylamidase (LeuA), Phosphatidylinositol phospholipase C (PIPLC), L-proline arylaminase (ProA), Tyrosine arylamidase (TyrA), L-aspartate arylaminase (AspA), β-glucuronidase (BGURr), β-galactopyranosidase (BGAR), α-galactosidase (AGAL), Urease (URE), β-galactosidase (BGAL), α-mannosidase (AMAN), Pyroglutamate arylaminase (PyrA), Phosphatase (PHOS) and β-D-glucuronidase (BGUR). Notably, only *W. confusa* RM125 could utilize xylose and tolerate Polymyxin B (POLYB), whereas only *W. cibaria* SJ548 could produce Alanine-phenylalanine-proline arylamidase (APPA) and Alanine arylamidase (AlaA). The results of Gram staining showed that *W. confusa* RM125 and *W. cibaria* SJ548 were Gram-positive bacteria (Supplementary Table S5).

#### 3.3.3 Antibacterial activity

The Oxford Cup method was employed to assess the inhibitory effects of *W. confusa* RM125 and *W. cibaria* SJ548 on eight indicator bacteria, revealing varying degrees of suppression exhibited by both strains. The distinct and nearly circular zones of inhibition were observed surrounding the Oxford cups (Figures 7C,D). The plates were then inspected to measure the diameter of the inhibition zone (DIZ). The DIZ was considered as follows: less than 15.00 mm indicated insensitivity; between 15.01 mm and 16.00 mm, it was classified as low sensitivity; between 16.01 mm and 17.00 mm, it was considered moderate sensitivity; and greater than 17.00 mm, it was classified as high sensitivity.

As illustrated in Table 1, *W. confusa* RM125 exhibited high sensitivity toward *V. harveyi*, moderate sensitivity toward *S. aureus* and *V. parahaemolyticus*, and low sensitivity toward *E. coli*, *A. hydrophila*, *S. enterica*, *V. anguillarum*, and *V. alginolyticus*. In contrast, *W. cibaria* SJ548 demonstrated high sensitivity toward both *V. harveyi* and *V. alginolyticus*, moderate sensitivity toward *A. hydrophila*, *S. aureus*, *S. enterica*, and *V. anguillarum*, and low sensitivity toward *E. coli* and *V. parahaemolyticus* (Table 1).

**Table 1:** The evaluation of antibacterial activity of *Weissella confusa* RM125 and *Weissella cibaria* SJ548, isolated from the midgut of mandarin fish fed an artificial diet, against eight indicator bacteria (*Escherichia coli*, *Aeromonas hydrophila*, *Staphylococcus aureus*, *Salmonella enterica*, *Vibrio anguillarum*, *Vibrio harveyi*, *Vibrio parahaemolyticus*, and *Vibrio alginolyticus*).

Indicator bacteria	RM125	SJ548
*Escherichia coli*	+	+
*Aeromonas hydrophila*	+	++
*Staphylococcus aureus*	++	++
*Salmonella enterica*	+	++
*Vibrio anguillarum*	+	++
*V. harveyi*	+++	+++
*V. parahaemolyticus*	++	+
*V. alginolyticus*	+	+++

Diameter of inhibition zone (DIZ) < 15.00 mm, insensitivity, no significance (ns); 15.00 mm < DIZ ≤ 16.00 mm, low sensitivity, +; 16.00 mm < DIZ ≤ 17.00 mm, moderate sensitivity, ++; DIZ > 17.00 mm, high sensitivity, +++.

## 4 Discussion

Gut is an essential tissue for digestion and absorption, of with its epithelial cells playing a crucial role in absorbing nutrients and distributing them throughout the body via the bloodstream to fulfill the organism’s material and nutritional requirements (Liu et al., 2023). Since mandarin fish are adapted to digesting live bait, we speculated that transformation of feeding habit in mandarin fish would have an impact on the structure and microbiota composition of gut. To investigate this, firstly, we conducted the histological observation and profile of microbiota, and then identified the dominant bacteria in the gut of mandarin fish fed either live bait or an artificial diet.

The height of intestinal villi is considered one of the most crucial markers of digestion and absorption (Suzuki et al., 2019). A reduction in villi height leads to a decrease in the surface area of the gut tract that comes into contact with digestive matter, thereby weakening the functions of absorption and digestion (Cao et al., 2022). Intestinal villi shortening is a key indicator of inflammation (Rysman et al., 2023), which may help explain the difficulty in transforming the feeding habit of mandarin fish. Previous studies have shown that feeding mandarin fish artificial diets may cause intestinal villi to shrink and rupture (Huang et al., 2023). Similar findings have been reported in Atlantic salmon (*Salmo salar*) and rainbow trout (*Oncorhynchus mykiss*) (Buttle et al., 2001). In the present study, the results of histological observation in gut revealed a significant decrease in intestinal villi height and muscle thickness in the foregut and midgut of mandarin fish fed artificial diets, consistent with the results reported by Chen et al. (2021). Furthermore, the muscle thickness of intestine and intestinal villi height showed a different tendency in the foregut, midgut, and hindgut, suggesting adaptive changes in the gut structure of mandarin fish in response to artificial diets for improved digestion and absorption.

In fish, gut microbiota can be influenced by the various compositions and characteristics of diets (Ringø et al., 2016). The change of diets firstly affected the diversity and abundance of gut microbiota which were dependent on the environment of gut (Sonnenburg and Bäckhed, 2016; Bi et al., 2023). The gut microbiome predicted functions help elucidate the physiological features and metabolism capability of mandarin fish with different diets. Furthermore, in the present study, we investigated the diversity of gut microbiota in mandarin fish fed with live bait and artificial diet. The results showed that there was no significant difference in Alpha diversity, but a significant difference in Beta diversity in gut bacterial communities between the two groups of mandarin fish. Previous reports have shown that feeding artificial diets can alter fish gut microbial richness and diversity (Hao et al., 2021). Firmicutes and Proteobacteria are the most prevalent and co-occurring bacterial taxa in the fish gut in terms of abundance of bacteria at the phylum classification level (Ingerslev et al., 2014), such as hairtail (*trichiurus haumela*), zebrafish, Nile tilapia (*Oreochromis niloticus*), and grass carp (*Ctenopharyngodon idella*) (Roeselers et al., 2011; Liu et al., 2017). In this study, Firmicutes and Proteobacteria were also the dominant phylum in the intestine of mandarin fish, which was consistent with Li′s research results (Li et al., 2017). At the genus level, the abundance of *Weissella* (67.70%) was significantly increased in the artificial feeding group, compared with the group fed with live bait, suggesting that the colonization of *Weissella* may be involved in the transformation of feeding habits in mandarin fish during domestication. Moreover, the analysis using PICRUSt2 revealed that the abundance of metabolic pathways, biosynthesis of secondary metabolites, microbial metabolism in diverse environments, and carbon metabolism in mandarin fish fed artificial diet were more abundant than those in the live fish bait. Previous studies have indicated that the gut microbiota of mandarin fish exhibited a high prevalence of metabolic functions, particularly in metabolic pathways such as chemoheterotrophy, fermentation, and nitrate reduction (Bourdichon et al., 2012). Though 16S rRNA sequencing is a great tool for taxonomic information, there are several limitations to such functional predictions, including strain level ambiguity within the identified species and dependency on the reference database. Additionally, PICRUSt2 cannot fully predict the metabolic capacity of microbial communities (Ortiz‐Estrada et al., 2019). Therefore, the metagenome of the gut could be used to perform the profound research in mandarin fish. Taken together, these findings suggest that mandarin fish possessed the capability to consume artificial feeds, break down and metabolize substantial quantities of amino acids, proteins, fats, and other essential nutrients present in the artificial diet.

*Weissella* has been reported to be a potential probiotic for use in poultry and aquatic animal feed (Sturino, 2018). In the present study, *W. confusa* RM125 and *W. cibaria* SJ548, belonging to *Weissella*, were isolated and identified from the midgut of mandarin fish fed with artificial diet. This finding was consistent with the results obtained from 16S rRNA sequencing. The biochemical information of these two strains was obtained by the VITEK2 method, revealing a high similarity in their utilization of carbon sources such as NAG, dMNE, SAC and dMAL. Furthermore, *W. confusa* and *W. cibaria* were reported to produce a high amount of glucan by hydrolyzing sucrose to glucose (Zeng and Burne, 2013). Our results indicated that only *W. confusa* RM125, and not *W. cibaria* SJ548, could utilize xylose. Notably, the ability to metabolize xylose could serve as a criterion to distinguish *W. confusa* from *W. cibaria*, thereby confirming the accuracy of our results in differentiating these two strains (Quattrini et al., 2020).

In addition, *Weissella* have been proven to possess antibacterial properties due to their production of extracellular polysaccharides, bacteriocins and organic acids (Kibar et al., 2020; Tenea and Lara, 2019; Teixeira et al., 2021). Based on the criteria investigated in this study, it was possible to isolate *Weissella* with their probiotic potential from the midgut of mandarin fish fed an artificial diet. Two strains *W. confusa* RM125 and *W. cibaria* SJ548 identified in our screening displayed a high potential for use as probiotics in aquaculture as they inhibited common aquaculture pathogens with a broad spectrum of bacteriostatic properties. Both *W. confusa* RM125 and *W. cibaria* SJ548 could effectively suppress a range of widespread bacteria in aquatic environments, including *E. coli*, *A. hydrophila*, *S. aureus*, *S. enterica*, and *V. anguillarum*, as well as those distributed specifically in seawater habitats, such as *V. harveyi*, *V. parahaemolyticus*, and *V. alginolyticus*, revealing a broad spectrum of bacteriostatic properties of these two strains. The similar broad inhibitory spectrum was found in *W. cibaria* HN05 and *W. cibaria* C-10 from Pacific white shrimp (*Litopenaeus vannamei*) and crucian carp (*Carassius auratus*), respectively, suggesting the probiotic potential of *Weissella* for aquaculture (Huy et al., 2020; Zhu et al., 2022). Besides, *Weissella* possessed antioxidant and bacteriostatic properties, facilitated digestion and absorption, implying its probiotic potential (Wang et al., 2020). Furthermore, several studies have found an enhancement immunity and improvement of feed conversion rate in aquatic animals by adding *Weissella* to the artificial diets, including crucian carp (*Carassius auratus*) (Zhu et al., 2022) and rainbow trout (*Oncorhynchus mykiss*) (Kahyani et al., 2021). These researches strongly suggested that *Weissella* possessed a potential probiotic characteristic. Taken together, the high abundance and probiotic properties of *W. confusa* RM125 and *W. cibaria* SJ548 illustrated that *Weissella* could benefit the digestion and absorption of the artificial diet in mandarin fish.

## 5 Conclusion

In the present study, we performed the histological observation, profile of microbiota and identification of dominant bacteria in gut of mandarin fish fed with live bait and artificial diet. The results of histological observation in gut showed that feeding with artificial diet showed a significant reduction in the intestinal villi height and the muscle thickness in the foregut and midgut, indicating adaptive changes in gut structure. The artificial diet significantly affected the diversity and abundance of gut microbiota in mandarin fish, with a significant increase in the abundance of *Weissella* (67.70%), indicating that the colonization of *Weissella* might be involved in the domestication and feeding habit transformation of mandarin fish. Furthermore, *W. confusa* RM125 and *W. cibaria* SJ548 with high abundance and probiotic properties were identified from the contents of gut in mandarin fish fed with artificial diet.

## Data Availability

The data presented in the study are deposited in the NCBI repository, accession number: PRJNA1171181 https://www.ncbi.nlm.nih.gov/bioproject/1171181.

## References

[ref1] Bi S. Yi H. Lai H. Li H. Liu X. Chen Q.. (2023). Intestinal microbiota of the four omnivorous fishes revealed by 16S rRNA metabarcoding from the habitats of oyster reefs. Ecol. Indic. 154:110895. doi: 10.1016/j.ecolind.2023.110895

[ref2] Bourdichon F. Casaregola S. Farrokh C. Frisvad J. C. Gerds M. L. Hammes W. P.. (2012). Food fermentations: microorganisms with technological beneficial use. Int. J. Food Microbiol. 154, 87–97. doi: 10.1016/j.ijfoodmicro.2011.12.030 22257932

[ref3] Brugman S. Ikeda-Ohtsubo W. Braber S. Folkerts G. Pieterse C. M. J. Bakker P. A. H. M. (2018). A comparative review on microbiota manipulation: lessons from fish, plants, livestock, and human research. Front. Nutr. 5:80. doi: 10.3389/fnut.2018.00080, PMID: 30234124 PMC6134018

[ref4] Buttle L. G. Burrells A. C. Good J. E. Williams P. D. Southgate P. J. Burrells C. (2001). The binding of soybean agglutinin (SBA) to the intestinal epithelium of Atlantic salmon, Salmo salar and rainbow trout, *Oncorhynchus mykiss*, fed high levels of soybean meal. Vet. Immunol. Immunopathol. 80, 237–244. doi: 10.1016/S0165-2427(01)00269-0, PMID: 11457477

[ref5] Cao K. Wang Y. Li M. Zhang C. Lahaye L. Chowdhury M. K.. (2022). Supplementation of a multienzyme complex, an organic acid-essential oil complex, and prebiotic alone or in combination affects growth, nutrient utilization, and immune function of rainbow trout. (*Oncorhynchus mykiss*). Aquacult. Nutr. 2022:1068537. doi: 10.1155/2022/1068537

[ref6] Chen X. Yi H. Liu S. Zhang Y. Su Y. Liu X.. (2021). Promotion of pellet-feed feeding in mandarin fish (*Siniperca chuatsi*) by *Bdellovibrio bacteriovorus* is influenced by immune and intestinal flora. Aquaculture 542:736864. doi: 10.1016/j.aquaculture.2021.736864

[ref7] Dou Y. Q. He S. Liang X. F. Cai W. J. Wang J. Shi L. J.. (2018). Memory function in feeding habit transformation of mandarin fish (*Siniperca chuatsi*). Int. J. Mol. Sci. 19:1254. doi: 10.3390/ijms19041254, PMID: 29690543 PMC5979507

[ref8] Hao Y. Sun Y. Li M. Fang X. Wang Z. Zuo J.. (2023). Adverse effects of polystyrene microplastics in the freshwater commercial fish, grass carp (*Ctenopharyngodon idella*): emphasis on physiological response and intestinal microbiome. Sci. Total Environ. 856:159270. doi: 10.1016/j.scitotenv.2022.159270, PMID: 36208741

[ref9] Hao Q. Teame T. Wu X. Ding Q. Ran C. Yang Y.. (2021). Influence of diet shift from bloodworm to formulated feed on growth performance, gut microbiota structure and function in early juvenile stages of hybrid sturgeon (*Acipenser baerii* × *Acipenser schrenckii*). Aquaculture 533:736165. doi: 10.1016/j.aquaculture.2020.736165

[ref10] Huang Y. Huang Q. Huang Z. Hong Y. (2024). Effects of formulated pellet feed or live fish food on the intestinal and aquaculture water microbial communities in goldfish, *Carassius auratus*. Water 16:2486. doi: 10.3390/w16172486

[ref11] Huang X. Yao X. Zheng J. Zhao J. (2023). Effects of compound feed on the intestinal structure and immune function of *Siniperca chuatsi*. Freshwater Fisheries 53, 77–85. doi: 10.13721/j.cnki.dsyy.2023.05.004

[ref12] Huy N. D. Ngoc L. M. T. Loc N. H. Lan T. T. Quang H. T. Dung T. (2020). Isolation of *Weissella cibaria* from Pacific white shrimp (*Litopenaeus vannamei*) gastrointestinal tract and evaluation of its pathogenic bacterial inhibition. IJST 13, 1200–1212. doi: 10.17485/ijst/2020/v13i10/149934

[ref13] Ingerslev H. C. von Gersdorff Jørgensen L. Lenz Strube M. Larsen N. Dalsgaard I. Boye M.. (2014). The development of the gut microbiota in rainbow trout (*Oncorhynchus mykiss*) is affected by first feeding and diet type. Aquaculture 424-425, 24–34. doi: 10.1016/j.aquaculture.2013.12.032

[ref14] Jin W. Jiang L. Hu S. Zhu A. (2024). Effects of lactobacillus plantarum and *Bacillus subtilis* on growth, immunity and intestinal flora of largemouth bass (*Micropterus salmoides*). Aquaculture 583:740581. doi: 10.1016/j.aquaculture.2024.740581

[ref15] Kahyani F. Pirali-Kheirabadi E. Shafiei S. Shenavar Masouleh A. (2021). Effect of dietary supplementation of potential Probiotic*weissella confusa* on innate immunity, immune‐related genes expression, intestinal microbiota and growth performance of rainbow trout (*Oncorhynchus mykiss*). Aquac. Nutr. 27, 1411–1420. doi: 10.1111/anu.13279

[ref16] Kibar H. Arslan Y. E. Ceylan A. Karaca B. Haliscelik O. Kiran F. (2020). *Weissella cibaria* EIR/P2-derived exopolysaccharide: a novel alternative to conventional biomaterials targeting periodontal regeneration. Int. J. Biol. Macromol. 165, 2900–2908. doi: 10.1016/j.ijbiomac.2020.10.106, PMID: 33736289

[ref17] Kotzamanis Y. P. Gisbert E. Gatesoupe F. J. Zambonino Infante J. Cahu C. (2007). Effects of different dietary levels of fish protein hydrolysates on growth, digestive enzymes, gut microbiota, and resistance to *Vibrio anguillarum* in European sea bass (*Dicentrarchus labrax*) larvae. Comp. Biochem. Physiol. A Physiol. 147, 205–214. doi: 10.1016/j.cbpa.2006.12.037, PMID: 17306580

[ref18] Kumar A. Augustine D. Sudhindran S. Kurian A. M. Dinesh K. R. Karim S.. (2011). *Weissella confusa*: a rare cause of vancomycin-resistant gram-positive bacteraemia. Int. J. Med. Microbiol. 60, 1539–1541. doi: 10.1099/jmm.0.027169-0, PMID: 21596906

[ref19] Li L. Fang J. Liang X. F. Alam M. S. Liu L. Yuan X. (2019). Effect of feeding stimulants on growth performance, feed intake and appetite regulation of mandarin fish, *Siniperca chuatsi*. Aquac. Res. 50, 3684–3691. doi: 10.1111/are.14327

[ref20] Li Y. Li J. Lu J. Li Z. Shi S. Liu Z. (2017). Effects of live and artificial feeds on the growth, digestion, immunity and intestinal microflora of mandarin fish hybrid (*Siniperca chuatsi*♀ × *Siniperca scherzeri*♂). Aquac. Res. 48, 4479–4485. doi: 10.1111/are.13273

[ref21] Li H. Niu S. Pan H. Wang G. Xie J. Tian J.. (2023). Integrated miRNA-mRNA analysis reveals the molecular mechanism in mandarin fish (*Siniperca chuatsi*) in response to fresh baits and artificial diets feeding. Aquac. Rep. 30:101554. doi: 10.1016/j.aqrep.2023.101554

[ref22] Li H. Zeng Y. Wang G. Zhang K. Gong W. Li Z.. (2024). Betaine improves appetite regulation and glucose-lipid metabolism in mandarin fish (*Siniperca chuatsi*) fed a high-carbohydrate-diet by regulating the AMPK/mTOR signaling. Heliyon 10:e28423. doi: 10.1016/j.heliyon.2024.e28423 38623237 PMC11016588

[ref23] Liang X. F. Oku H. Ogata H. Y. Liu J. He X. J. A. R. (2001). Weaning Chinese Perch*siniperca chuatsi* (Basilewsky) onto artificial diets based upon its specific sensory modality in feeding. Aquac. Res. 32, 76–82. doi: 10.1046/j.1355-557x.2001.00006.x

[ref24] Ligozzi M. Bernini C. Bonora M. G. De Fatima M. Zuliani J. Fontana R. (2002). Evaluation of the VITEK2 system for identification and antimicrobial susceptibility testing of medically relevant gram-positive cocci. J. Clin. Microbiol. 40, 1681–1686. doi: 10.1128/JCM.40.5.1681-1686.2002, PMID: 11980942 PMC130656

[ref25] Liu Y. Li X. Li J. Chen W. (2021). The gut microbiome composition and degradation enzymes activity of black Amur bream (*Megalobrama terminalis*) in response to breeding migratory behavior. Ecol. Evol. 11, 5150–5163. doi: 10.1002/ece3.7407, PMID: 34025998 PMC8131771

[ref26] Liu H. Pan L. Shen J. Tan B. Dong X. Yang Q.. (2023). Effects of carbohydrase supplementation on growth performance, intestinal digestive enzymes and Flora, glucose metabolism enzymes, and glut2 gene expression of hybrid grouper (*Epinephelus fuscoguttatus*♀ × *E. lanceolatus*♂) fed different CHO/L ratio diets. Meta 13:98. doi: 10.3390/metabo13010098, PMID: 36677024 PMC9865975

[ref27] Liu W. S. Wang W. W. Ran C. He S. X. Yang Y. L. Zhou Z. G. (2017). Effects of dietary scFOS and lactobacilli on survival, growth, and disease resistance of hybrid tilapia. Aquaculture 470, 50–55. doi: 10.1016/j.aquaculture.2016.12.013

[ref28] Ma R. Wang Y. Zhao S. Ma Q. Yin M. Li X.. (2021). Bacterial Flora in the gill tissues and intestinal tracts of male and female Chinese mitten crabs (*Eriocheir sinensis*) with different diets in a mud pond. Curr. Microbiol. 78, 2291–2297. doi: 10.1007/s00284-021-02487-9, PMID: 33860342

[ref7000] Ortiz‐Estrada Á. M. Gollas‐Galván T. Martínez‐Córdova L. R. Martínez‐Porchas M.. (2019). Predictive functional profiles using metagenomic 16S rRNA data: a novel approach to understanding the microbial ecology of aquaculture systems. Rev. Aquacult. 11, 234–245., PMID: 33860342

[ref29] Quattrini M. Korcari D. Ricci G. Fortina M. G. (2020). A polyphasic approach to characterizeWeissella Cibariaand*weissella confusa*strains. Lett. Appl. Microbiol. 128, 500–512. doi: 10.1111/jam.14483 31602728

[ref30] Ray A. K. Ghosh K. Ringø E. J. A. N. (2012). Enzyme - producing bacteria isolated from fish gut: a review. Aquac. Nutr. 18, 465–492. doi: 10.1111/j.1365-2095.2012.00943.x

[ref31] Ringø E. Z. Z. V. Zhou Z. Vecino J. G. Wadsworth S. Romero J. Krogdahl A.. (2016). Effect of dietary components on the gut microbiota of aquatic animals. A never - ending story? Aquac. Nutr. 22, 219–282. doi: 10.1111/anu.12346

[ref32] Roeselers G. Mittge E. K. Stephens W. Z. Parichy D. M. Cavanaugh C. M. Guillemin K.. (2011). Evidence for a core gut microbiota in the zebrafish. ISME J. 5, 1595–1608. doi: 10.1038/ismej.2011.38, PMID: 21472014 PMC3176511

[ref33] Rysman K. Eeckhaut V. Ducatelle R. Goossens E. Van Immerseel F. (2023). Broiler performance correlates with gut morphology and intestinal inflammation under field conditions. Avian Pathol. 52, 232–241. doi: 10.1080/03079457.2023.2201169, PMID: 37132444

[ref34] Sambrook J. Russell D. W. (2001). Molecular cloning: a laboratory manual. 3rd Edn: New York: Cold Spring Harbor Laboratory Press.

[ref35] Shen Y. Li H. Zhao J. Tang S. Zhao Y. Bi Y.. (2021). The digestive system of mandarin fish (*Siniperca chuatsi*) can adapt to domestication by feeding with artificial diet. Aquaculture 538:736546. doi: 10.1016/j.aquaculture.2021.736546

[ref36] Shen J. Liu H. Tan B. Dong X. Yang Q. Chi S.. (2020). Effects of replacement of fishmeal with cottonseed protein concentrate on the growth, intestinal microflora, haematological and antioxidant indices of juvenile golden pompano (*Trachinotus ovatus*). Aquac. Nutr. 26, 1119–1130. doi: 10.1111/anu.13069

[ref37] Shen Y. Song L. Chen T. (2023). Identification of hub genes in digestive system of mandarin fish (*Siniperca chuatsi*) fed with artificial diet by weighted gene co-expression network analysis. Comp. Biochem. Physiol. D Genom. Proteom. 47:101112. doi: 10.1016/j.cbd.2023.101112 37516099

[ref38] Shi B. Qian T. Yin Z. Zhang Y. Feng T. Dong Z.. (2024). Comparing effects of high starch diet or high lipid diet supplemented with different levels of zinc on intestinal barrier and microbe community in largemouth bass *Micropterus salmoides*. Fish Shellfish Immunol. Rep. 154:109911. doi: 10.1016/j.fsi.2024.109911, PMID: 39293705

[ref39] Sonnenburg J. L. Bäckhed F. (2016). Diet - microbiota interactions as moderators of human metabolism. Nature 535, 56–64. doi: 10.1038/nature18846, PMID: 27383980 PMC5991619

[ref40] Sturino J. M. (2018). Literature-based safety assessment of an agriculture-and animal-associated microorganism: *Weissella confusa*. Regul. Toxicol. Pharmacol. 95, 142–152. doi: 10.1016/j.yrtph.2018.03.013, PMID: 29567328

[ref41] Suzuki T. Mayanagi Y. Keta A. Kasahara A. Sato A. Takahashi T. (2019). Oral administration of fructose improves jejunal villous morphology and nutrient digestion and absorption capabilities in a rat model of total parenteral nutrition. Biomed. Res. Clin. Pract. 4, 1–8. doi: 10.15761/BRCP.1000179

[ref42] Tao J. Gong Y. Chen S. Li W. Xie R. Zhang H.. (2024). Dietary inclusion of *Clostridium butyricum* cultures alleviated impacts of high-carbohydrate diets in largemouth bass (*Micropterus salmoides*). Brit. J. Nutr 131, 1308–1325. doi: 10.1017/S0007114523002842, PMID: 38073302

[ref43] Teixeira C. G. Fusieger A. Martins E. de Freitas R. Vakarelova M. Nero L. A.. (2021). Biodiversity and technological features of Weissella isolates obtained from Brazilian artisanal cheese-producing regions. LWT 147:111474:111474. doi: 10.1016/j.lwt.2021.111474

[ref44] Tenea G. N. Lara M. I. (2019). Antimicrobial compounds produced By*weissella confusa*cys2-2 strain inhibit gram-negative bacteria growth. CyTA J. Food 17, 105–111. doi: 10.1080/19476337.2018.1561520

[ref45] Vargas-Albores F. Martínez-Córdova L. R. Hernández-Mendoza A. Cicala F. Lago-Lestón A. Martínez-Porchas M. (2021). Therapeutic modulation of fish gut microbiota, a feasible strategy for aquaculture? Aquaculture 544:737050. doi: 10.1016/j.aquaculture.2021.737050

[ref46] Vargas-González A. Barajas M. Pérez-Sánchez T. (2024). Isolation of lactic acid Bacteria (LAB) from salmonids for potential use as probiotics: in vitro assays and toxicity assessment of *Salmo trutta* Embryonated eggs. Animals 14:200. doi: 10.3390/ani14020200, PMID: 38254369 PMC10812622

[ref47] Vine N. G. Leukes W. D. Kaiser H. Daya S. Baxter J. Hecht T. (2004). Competition for attachment of aquaculture candidate probiotic and pathogenic bacteria on fish intestinal mucus. J. Fish Dis. 27, 319–326. doi: 10.1111/j.1365-2761.2004.00542.x, PMID: 15189372

[ref49] Wang W. Liu W. Chu W. (2020). Isolation and preliminary screening of potentially probiotic *Weissella confusa* strains from healthy human feces by culturomics. Microb. Pathog. 147:104356. doi: 10.1016/j.micpath.2020.104356, PMID: 32610159

[ref50] Wang J. Zhang L. Gao X. Sun Y. Zhao C. Gao X.. (2024). Molecular cloning of the *scd1* gene and its expression in response to feeding artificial diets to mandarin fish (*Siniperca chuatsi*). Genes 15:1211. doi: 10.3390/genes15091211, PMID: 39336802 PMC11431013

[ref51] Wei H. Tan B. Yang Q. Mao M. Lin Y. Chi S. (2023). Growth, nonspecific immunity, intestinal flora, hepatopancreas, and intestinal histological results for *Litopenaeus vannamei* fed with diets supplement with different animal by-products. Aquac. Rep. 29:101521. doi: 10.1016/j.aqrep.2023.101521

[ref52] Xu Y. H. Wei X. L. Xu Y. C. Zhang D. G. Zhao T. Zheng H.. (2022). Waterborne enrofloxacin exposure activated oxidative stress and MAPK pathway, induced apoptosis and resulted in immune dysfunction in the gills of yellow catfish *Pelteobagrus fulvidraco*. Aquaculture 547:737541. doi: 10.1016/j.aquaculture.2021.737541

[ref53] Yadav M. K. Tiwari S. K. (2023). Methods for determination of antimicrobial activity of bacteriocins of lactic acid bacteria. Microbiology 92, 745–765. doi: 10.1134/S0026261723600520

[ref54] Yang B. Song H. Hu R. Tao L. Liang Z. Cong W.. (2023). *Weissella confusa* N17 derived from loach (*Misgurnus anguillicaudatus*) exhibits promising for further applications in loach aquaculture. Probiot. Antimicrob. Prot., 1–15. doi: 10.1007/s12602-023-10149-4, PMID: 37632675

[ref55] Zeng L. Burne R. A. (2013). Comprehensive mutational analysis of sucrose-metabolizing pathways in *Streptococcus mutans* reveals novel roles for the sucrose phosphotransferase system permease. J. Bacteriol. 195, 833–843. doi: 10.1128/JB.02042-12, PMID: 23222725 PMC3562097

[ref56] Zhang L. H. Luo Z. Song Y. F. Shi X. Pan Y. X. Fan Y. F.. (2016). Effects and mechanisms of waterborne copper exposure influencing ovary development and related hormones secretion in yellow catfish *Pelteobagrus fulvidraco*. Aquat. Toxicol. 178, 88–98. doi: 10.1016/j.aquatox.2016.07.014, PMID: 27472784

[ref57] Zhang Z. Yuan X. Wu H. Gao J. Wu J. Xiong Z.. (2024). The effect of short-term artificial feed domestication on the expression of oxidative-stress-related genes and antioxidant capacity in the liver and gill tissues of mandarin fish (*Siniperca chuatsi*). Genes 15:487. doi: 10.3390/genes15040487, PMID: 38674421 PMC11050011

[ref58] Zhu X. K. Yang B. T. Hao Z. P. Li H. Z. Cong W. Kang Y. H. (2022). Dietary supplementation with *Weissella cibaria* C-10 and *Bacillus amyloliquefaciens* T-5 enhance immunity against *Aeromonas veronii* infection in crucian carp (*Carassiu auratus*). Microb. Pathog. 167:105559. doi: 10.1016/j.micpath.2022.105559, PMID: 35568093

