# Supplementary material for: Profiling the gut structure and microbiota, and identifying two dominant bacteria belonging to the Weissella genus in mandarin fish (Siniperca chuatsi) fed an artificial diet
Source: Front Microbiol. 2024 Nov 29;15:1486501. doi: 10.3389/fmicb.2024.1486501 (PMC11639983; doi:10.3389/fmicb.2024.1486501)
Supplement: Supplementary file 1 [file Supplementary_file_1.docx]

**Supplementary Table 1** The nutrient composition of the artificial diets for mandarin fish.

| Nutritional composition | Percentages |
| --- | --- |
| crude protein | ≥ 48.0% |
| crude fat | ≥ 10.0% |
| crude fiber | ≤ 4.0% |
| crude ash | ≤ 12.0% |
| lysine | ≥ 2.8% |
| calcium | ≥ 1.5% |
| total phosphorus | ≥ 1.5% |
| moisture | ≤ 12.0% |

**Supplementary Table 2** The sequence information, diversity, and richness indices of the gut microbiota population in mandarin fish with different feeding groups (C and T).

| Sample | Term | Diversity indices | | Richness indices | |
| --- | --- | --- | --- | --- | --- |
|  |  | Shannon index | Simpson index | Ace index | Chao1 index |
| C | 300726 | 3.352±1.319 | 0.708±0.100 | 279.00±238.50 | 279.00±238.50 |
| T | 341285 | 3.865±0.996 | 0.710±0.090 | 437.25±192.44 | 437.25±192.44 |

Data were presented as mean± SEM. Abbreviation: ACE: abundance-based coverage estimator; C, live fish bait group; T, artificial feed group. *****: *p* < 0.05, a significant difference; ******: *p* < 0.01, extremely significant difference; ns, no significant difference.

**Supplementary Table 3** The abundance of species in the artificial diet at the level of bacterial genera.

| Bacterial species abundance | Percentages |
| --- | --- |
| *Pantoea* | 18.09% |
| *Bacillus* | 15.20% |
| *Chryseobacterium* | 9.99% |
| *Planococcus* | 6.23% |
| *Pseudomonas* | 4.70% |
| Other | ≥11.22% |
| unidentified_Mitochondria | 20.66% |
| unidentified_Chloroplast | 13.91% |

**Supplementary Table 4** The KEGG metabolic pathway associated with gut microbiota genes in different feeding groups (C and T) at Level 1.

| Function | T | C |
| --- | --- | --- |
| Metabolism | 139,731,136 | 536,937,850 |
| Human Diseases | 9,414,725 | 19,144,145 |
| Genetic Information Processing | 25,386,603 | 59,904,308 |
| Environmental Information Processing | 21,963,361 | 60,829,254 |
| Cellular Processes | 9,233,093 | 28,426,623 |
| Organismal Systems | 5,022,086 | 10,550,972 |

Abbreviations: C, Control group (live bait group); T, Treatment group (artificial feed group).

**Supplementary Table 5** Physiological and biochemical characteristics of bacteria RM125 and SJ548 isolated and identified from the midgut of mandarin fish fed with artificial diet.

| Item | RM125 | SJ548 | Item | RM125 | SJ548 | Item | RM125 | SJ548 |
| --- | --- | --- | --- | --- | --- | --- | --- | --- |
| AMY | － | － | dXYL | ＋ | － | AMAN | ＋ | － |
| APPA | － | ＋ | AspA | － | － | PyrA | ＋ | ＋ |
| LeuA | － | － | BGURr | － | － | POLYB | － | － |
| AlaA | － | ＋ | dSOR | － | － | dMAL | － | － |
| dRIB | － | － | LAC | － | － | MBdG | － | － |
| NOVO | ＋ | ＋ | dMAN | － | － | dTRE | － | － |
| dRAF | － | － | SAL | － | － | AGLU | － | － |
| OPTO | ＋ | ＋ | ADH1 | ＋ | ＋ | PHOS | － | － |
| PIPLC | － | － | BGAR | － | － | BGUR | ＋ | ＋ |
| CDEX | － | － | AGAL | － | － | dGAL | － | － |
| ProA | － | － | URE | － | － | BACI | ＋ | ＋ |
| TyrA | － | － | NAG | ＋ | ＋ | PUL | － | － |
| NC6.5 | ＋ | ＋ | dMNE | ＋ | ＋ | ADH2s | ＋ | ＋ |
| O129R | ＋ | ＋ | SAC | － | － | BGAL | － | － |
| Gram staining | ＋ | ＋ |  |  |  |  |  |  |

Abbreviations: AMY, Amygdalin; APPA, Alanine-phenylalanine-proline arylamidase; LeuA, Leucine arylamidase; AlaA, Alanine arylamidase; dRIB, d-ribose; NOVO, Novobiocin tolerance; dRAF, D-raffinose; OPTO, Optokhin tolerance; PIPLC, Phosphatidylphospholipase C; CDEX, Cyclodextrin; ProA, L-proline arylaminase; TyrA, Tyrosine arylamidase; NC6.5, 6.5%NaCl growth; O129R, O/129 tolerance; ADH2s, Arginine dihydrolase 2; dXYL, D-xylose; AspA, L-aspartate arylaminase; BGURr, β-glucuronidase; dSOR, D-sorbitol; LAC, Lactose; dMAN, D-mannitol; SAL, Salicin; ADH1, Arginine dihydrolase 1; BGAR, β-galactopyranosidase; AGAL, α-galactosidase; URE, Urease; NAG, N-acetyl-D-glucosamine; dMNE, D-mannose; SAC, Saccharose; BGAl, β-galactosidase; AMAN, α-mannosidase; PyrA, Pyroglutamate arylaminase; POLYB, Polymyxin B tolerance; dMAL, D-maltose; MBdG, Methyl-B-D-glucopyranoside; dTRE, D-trehalose; AGLU, α-glucosidase; PHOS, Phosphatase; BGUR, β-D-glucuronidase; dGAL, D-galactose; BACI, Bacitracin tolerance; PUL, Pullulanase.


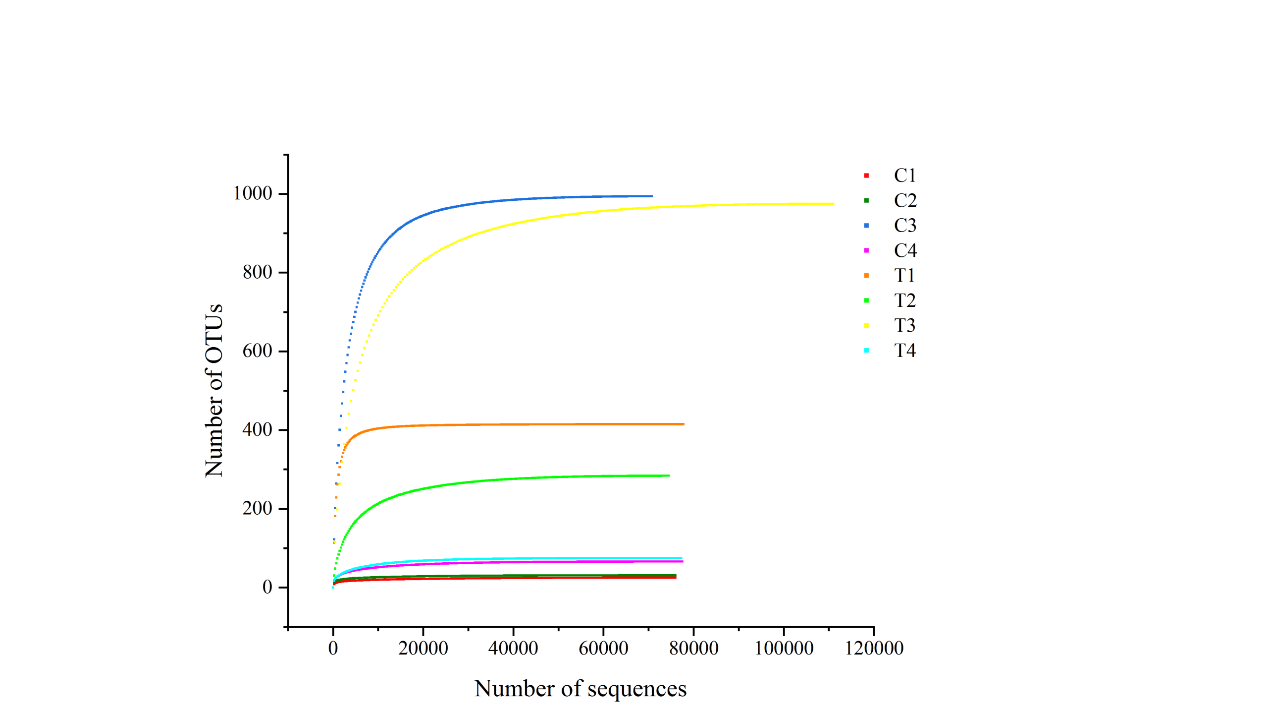


**Supplementary Figure 1** Rarefaction curves of 8 samples of mandarin fish in different feeding groups (C and T) after feeding for 8 weeks. Abbreviation: C, live bait group; T, artificial diet group.


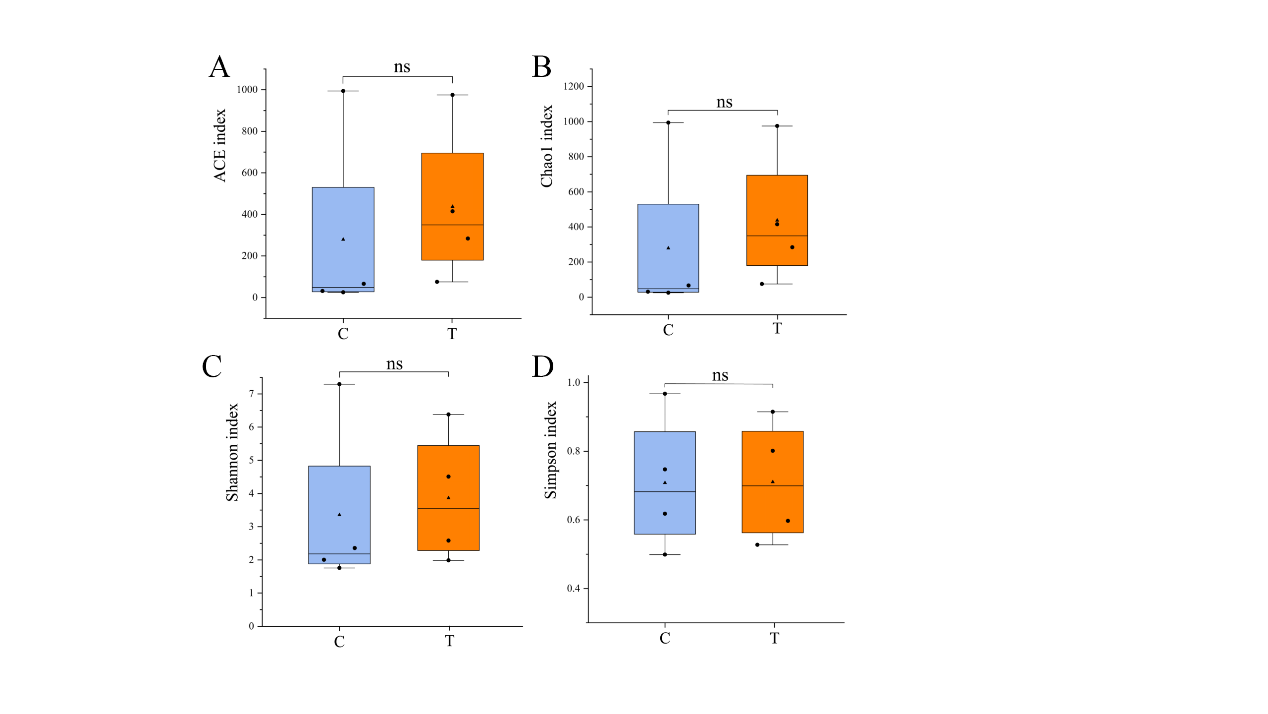


**Supplementary Figure 2** The Alpha diversity index of intestinal samples of mandarin fish in different feeding groups (C and T) after feeding for 8 weeks. ACE index (A); Chao 1 index (B); Shonnon index (C); Simpson index (D). Abbreviation: C, live bait group; T, artificial diet group.

**Supplementary Figure 3** ClustalX alignment of the 16s rRNA sequences of *Weissella confusa* RM125 and other *Weissella*. The identical sequences were shaded dark grey. Accession numbers: *Weissella confusa* strain RM125, PP125780.1; *Weissella confusa* strain 6400, MT515841.1; *Weissella confusa* strain 2879, MT611841.1.

**Supplementary Figure 4** ClustalX alignment of the 16s rRNA sequences of *Weissella cibaria* SJ548 and other *Weissella*. The identical sequences were shaded dark grey. Accession numbers: *Weissella cibaria* strain SJ548, PP068943.1; *Weissella cibaria* strain 1382, MT611777.1; *Weissella cibaria* strain 2769, MT573803.1.
